# Encoding scheme design for gradient-free, nonlinear projection imaging using Bloch-Siegert RF spatial encoding in a low-field, open MRI system

**DOI:** 10.1038/s41598-024-53703-y

**Published:** 2024-02-08

**Authors:** Kartiga Selvaganesan, Yonghyun Ha, Heng Sun, Zhehong Zhang, Chenhao Sun, Anja Samardzija, Gigi Galiana, R. Todd Constable

**Affiliations:** 1 Department of Biomedical Engineering, Yale University, New Haven, CT USA; 2 Department of Radiology and Biomedical Imaging, Yale School of Medicine, New Haven, CT USA; 3 Yale University School of Medicine, Magnetic Resonance Research Center, 300 Cedar Street, New Haven, CT 06520 USA

**Keywords:** Magnetic resonance imaging, Biomedical engineering

## Abstract

Eliminating conventional pulsed B_0_-gradient coils for magnetic resonance imaging (MRI) can significantly reduce the cost of and increase access to these devices. Phase shifts induced by the Bloch-Siegert shift effect have been proposed as a means for gradient-free, RF spatial encoding for low-field MR imaging. However, nonlinear phasor patterns like those generated from loop coils have not been systematically studied in the context of 2D spatial encoding. This work presents an optimization algorithm to select an efficient encoding trajectory among the nonlinear patterns achievable with a given hardware setup. Performance of encoding trajectories or projections was evaluated through simulated and experimental image reconstructions. Results show that the encodings schemes designed by this algorithm provide more efficient spatial encoding than comparison encoding sets, and the method produces images with the predicted spatial resolution and minimal artifacts. Overall, the work demonstrates the feasibility of performing 2D gradient-free, low-field imaging using the Bloch-Siegert shift which is an important step towards creating low-cost, point-of-care MR systems.

## Introduction

Magnetic resonance imaging (MRI) has the advantage of imaging with non-ionizing radiation and providing superior soft tissue contrast, making it a valuable tool for disease diagnosis and treatment. Recently, there has been a push towards low-field MRI to reduce the cost of and increase access to the technology^1–3^. Much of the cost incurred by MR systems comes from the purchase, installation and maintenance of the magnet, cryostat cooling systems, gradient amplifiers, and gradient coils^1^. Attempts at reducing magnet costs have primarily focused on reimagining the conventional superconducting magnets and improving portability by implementing permanent magnet arrays to generate the main magnetic field. These systems use linear B_0_-gradients for spatial encoding, coupled with artificial intelligence techniques for image reconstruction^4–6^.

Other efforts have aimed to provide lower cost alternatives to B_0_-gradients. Eliminating gradient coils can significantly reduce the maintenance and operational costs of MRI. Moreover, gradient coils for producing linear x, y, and z gradient shapes, tend to have a cylindrical geometry which takes up room in the bore, and adds to the claustrophobic feeling some patients experience. This tunnel-like configuration in combination with the rapidly changing currents used to drive gradient coils generates high levels of acoustic noise^7^. Therefore, gradient-free spatial encoding technologies could not only reduce the financial burden of MRI, but also lead to a silent, open magnet system with increased patient comfort.

Previous work on radiofrequency-based^8^ spatial encoding techniques include rotating frame zeugmatography^9^, transmit array spatial encoding (TRASE)^10^, selective encoding through nutation and fingerprinting^11,12^, and frequency-modulated Rabi-encoding echoes (FREE)^13^. Rotating frame zeugmatography and TRASE imaging both perform RF encoding with specially designed coils that impute linear B_1_-field variations. The former requires a linear B_1_ magnitude gradient, while the latter needs a linear B_1_ phase gradient. While these techniques have shown promise in being able to perform spatial encoding with RF in at least one of the three Cartesian directions, they all focused on linearly varying B_1_-fields and a small field-of-view (FOV). RF coils inherently produce nonlinear fields so the design of these coils would be much simpler, and spatial encoding could be done over a larger FOV if the linearity constraint is eliminated. SENF and FREE imaging could potentially be done with nonlinear fields, however recent studies involving these methods have only employed encoding hardware and image acquisitions techniques involving linear B_1_-fields. Katscher et al. did show the use of nonlinear RF transmit fields for spatial encoding, however their multi-element parallel transmit system followed the conventional closed-bore magnet design^14^.

One way to perform gradient-free encoding while still maintaining the open magnet geometry is by encoding with the Bloch-Siegert shift using an RF planar array. Studies have demonstrated using the Bloch-Siegert phase shift in combination with existing imaging techniques for B_1_-field mapping^15,16^, but few have shown its use in spatially encoding MR signal. Those who have, limited themselves to linearly varying B_1_-encoding fields and imaging over a small FOV^17–19^. These studies also used the Bloch-Siegert shift in combination with conventional gradient coils to perform 2D spatial encoding.

In this study we propose methodologies for identifying efficient nonlinear encoding schemes for gradient-free imaging at low-field using the Bloch-Siegert shift. We analyze the effect of Bloch-Siegert pulse phase and number of transmit coils on the design of spatial encoding magnetic fields (SEM), as well as propose a robust optimization technique to select an optimal set of projections. Encoding schemes were evaluated with both simulated and experimental image reconstructions. The encoding technique proposed in this work can be translated to coil arrays of any number of channels, geometry, or orientation thereby demonstrating proof-of-principle of how planar RF array coils can be used for gradient-free spatial encoding and imaging with the Bloch-Siegert shift.

## Theory

### Nonlinear RF encoding with the Bloch-Siegert shift

The Bloch-Siegert shift effect is a phenomenon in which an off-resonance RF pulse (${\omega }_{RF}$) induces a frequency shift to the Larmor frequency (${\omega }_{0}$)^20^. Under the assumption that the relative off-resonance is much larger than the nutation frequency associated with the magnitude of the RF-field (B_1_ + -field),

1 $$\left({\omega }_{0}-{\omega }_{RF}\right)\gg \gamma {B}_{1}^{+}$$

and the constraint that the inherent field inhomogeneities $(\Delta {\omega }_{0})$ are negligible compared to the off-resonance frequencies,

2 $$\left({\omega }_{0}-{\omega }_{RF}\right)\gg \Delta {\omega }_{0}$$

the Bloch-Siegert shift is strictly a function of the B_1_ power. Thus the resulting phase shift is given by^16^:

3 $${\phi }_{BS}= {\int }_{0}^{\tau }\frac{{|\gamma B1|}^{2}}{2\Delta {\omega }_{RF}}dt$$

where $\Delta {\omega }_{RF}={\omega }_{0}-{\omega }_{RF}$, and $\gamma$ is the gyromagnetic ratio. Equation (3) can be derived by viewing the spins in the rotating frame of the effective B_1_-field. In this frame of reference, the off-resonance B_1_ is equivalent to the return of residual z-magnetization. Thus the effective B_1_-field is about a tilted axis that results from the transverse B_1_-field and the longitudinal off-resonance B_1_^21,22^.

If the B_1_-field is spatially varying, this leads to a spatial dependence in the phase shift and Eq. (3) becomes:

4 $${\phi }_{BS}\left(x,y\right)= {\int }_{0}^{\tau }\frac{{\left|\gamma B1\left(x,y\right)\right|}^{2}}{2\Delta {\omega }_{RF}}dt$$

Therefore, knowledge of the spatial variations in the B_1_-field could be intentionally exploited to encode MR signal. Such spatially varying B_1_-fields can be created with RF planar arrays. By selecting different combinations of coil elements from an RF array to transmit the off-resonance Bloch-Siegert pulse, one can produce unique field patterns similar to the encoding patterns produced by nonlinear B_0_-gradient fields^23,24^.

Since the encoding fields are proportional to |B_1_|^2^ a nonlinear phase shift is introduced over the imaging FOV, so Fourier transform-based image reconstruction algorithms will not work. In this case, a 1D Fourier transform of the readout signal will yield a projection of the object where points along the projection is the amount of excited magnetization along isocontour lines in the nonlinear B_1_-field—a process similar to the theory behind O-space imaging^25^. To obtain an undistorted image, projection-based reconstruction algorithms adapted to include the spatial dependence of the B_1_+-field are needed, like the conjugate gradient or algebraic reconstruction technique^26,27^. Ultimately, this method of encoding and reconstruction can yield a gradient-free, silent imaging approach to MRI.

## Methods

### Low-field magnet design

**Figure 1:**
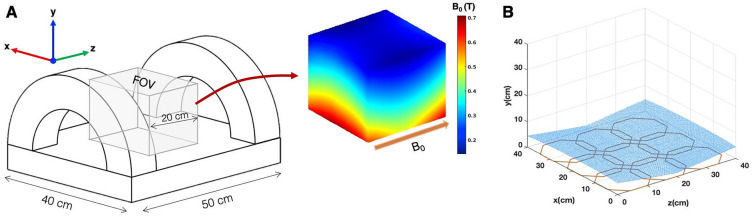
Low-field magnet system: (**A**) Design of the two-element electromagnet generating the main magnetic (B_0_-field) of the open MR system. The FOV of interest is a 20 cm × 20 cm region in the center of the magnet. The inset shows the measured B_0_-field map in this cubic FOV. The main magnetic field direction is from left to right. This field also serves as the slice select gradient, and (**B**) shows an example of the nonplanar imaging slice selected from a region of uniform magnetic field strength.

Figure 1A shows a schematic of the open low-field MRI system developed at the Yale Magnetic Resonance Research Center (MRRC). The main magnetic field is generated by two hollow copper coil elements that create a non-uniform magnetic field aligned to the anatomical left–right direction, and the gradient field in the anterior–posterior direction. The magnet is cooled with deionized water running through the hollow copper wires. Since the system is a rampable electromagnet, it can be dynamically controlled for both spin polarization and slice selection. The inset in Fig. 1A shows the B_0_-field pattern in a 20 cm × 20 cm region in the center of the FOV when the spins are maximally polarized. The magnitude of the B_0_-field through the center of the FOV ranges from 0.6 T at the base of the magnet to 0.2 T at a 20 cm depth. For imaging, spins are first maximally polarized with the magnet in high current mode. Then the magnet is ramped down until the desired slice is at 24 mT (1 MHz)—on resonance with the excitation and readout electronics at 1 MHz. It takes about 35 ms to ramp the magnet up or down. Figure 1B shows an example of a non-planar 24 mT imaging slice; the location of the slice is selected through current modulation, using the non-uniform B_0_ as a slice-select gradient.

### RF Encoding with the Bloch-Siegert shift

**Figure 2:**
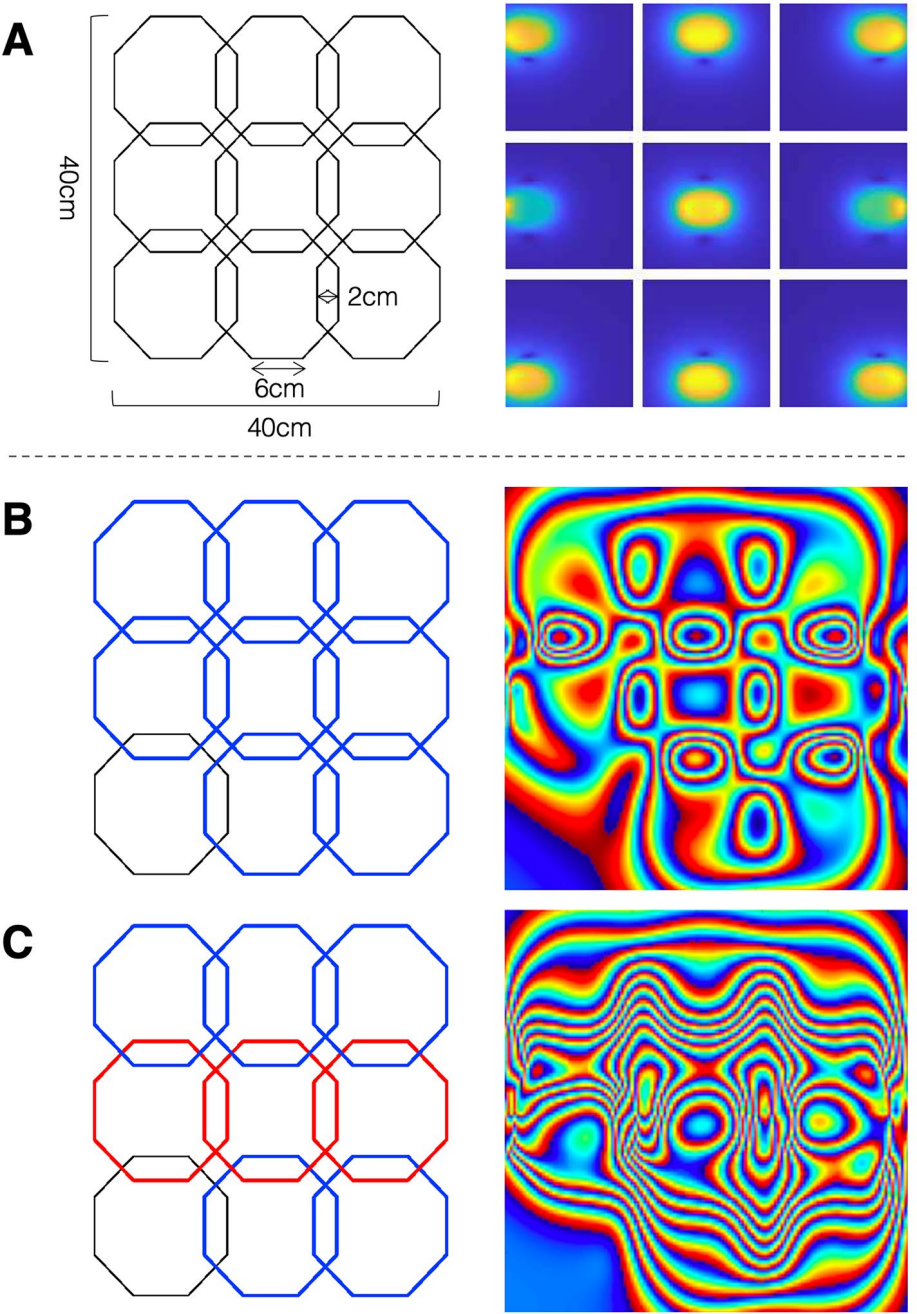
Phasor patterns. (**A**) Diagram showing the 9-channel RF array used to perform spatial encoding using the Bloch-Siegert shift and normalized coil sensitivity profiles across a single slice, calculated with the Biot–Savart law for each coil element. These fields were used to calculate the phasor patterns. (**B**) and (**C**) show examples of two different phasors generated from transmitting the Bloch-Siegert pulse on 8 coils where all channels are in phase or the middle row of transmit channels is $\pi$ out of phase, respectively.

With the B_0_-field in the z-direction (left–right), the RF encoding coils need to be built such that their B_1_-fields are oriented in the x- (superior–inferior) and y-directions (anterior–posterior). To satisfy this constraint while maintaining the open magnet design, a 9-channel planar RF array coil was created for Bloch-Siegert shift based encoding. The array covers the entire 40 cm × 40 cm area at the base of the magnet, with each octagonal coil element having side lengths of 6 cm and an overlap of 2 cm (Fig. 2A). The coil channels are switched tuned so that they are able to transmit at the off-resonance Bloch-Siegert frequency of 870 kHz and receive at the on-resonance frequency of 1 MHz.

At low-field strengths, the B_1_-transmit fields generated by RF coils can be estimated using quasistatic approximations because the RF coils are small compared to the wavelength of the RF inside biological tissue, so coil tissue interactions are negligible^28^. Therefore, the encoding B_1_-field may be calculated using the Biot–Savart Law. In this study, the B_1_-field generated by each element in the RF coil array was calculated in MATLAB using the Biot–Savart Magnetic Toolbox^29^. The toolbox performs a numerical integration of the Biot–Savart law to determine the magnetic flux density of any current carrying filament using the equation:

5 $$\overrightarrow{dB}= \frac{{\mu }_{0}}{4\pi }\frac{Id\overrightarrow{L }\times \widehat{r}}{{r}^{2}}$$

where, ${\mu }_{0}$ the permeability of free space, $d\overrightarrow{L}$ is infinitesimal length of a wire segment, $I$ is the current intensity, $r$ is the distance to a point in space, and $\widehat{r}$ is the unit vector describing the direction of that point from the coil. Figure 2A shows the magnitude coil sensitivity profiles of all nine coils in the 9-channel array across the nonplanar imaging slice, calculated using the Biot–Savart law. Only the x and y-components of the B_1_-field, those orthogonal to the main magnetic field, were considered in calculations of the receiver sensitivity profiles and encoding fields patterns.

Using these B1-fields, the encoding patterns ${({\text{B}}1}_{{\text{E}}})$ were calculated with the following equation:

6 $$B1_{E} = \sum\limits_{1}^{{c = 9}} {B1_{c} *A_{c} e^{{\left( { - i*\varphi _{c} } \right)}} }$$

In this equation ${\text{B}}{1}_{{\text{c}}}$ is the B_1_-field of each channel over the imaging slice. ${A}_{c}$ denotes the amplitude and ${\varphi }_{c}$, the phase of the Bloch-Siegert off-resonance applied to each channel in the RF encoding array*.* Maximum power from the RF power amplifier was applied to each channel; because of slight differences in coil matching conditions, the actual field generated by each channel was variable, so ${A}_{c}$ was scaled by an experimental measurement of the B_1_-field taken at a single point in the center of the coil at 5% of max power. Different encoding patterns can be created by varying the number of coils selected to transmit the Bloch-Siegert pulse, either 3, 4, 5 etc. These patterns can be thought to be analogous to nonlinear encoding fields generated from conventional gradient fields. Phasors, which refer to the state of magnetization at a single point in time, were computed using a Bloch simulation algorithm commonly described in the literature^30,31^. The Bloch simulations used Cayley-Klein parameters to represent the rotation caused by the off-resonance RF pulse on isochromats over time.

Figure 2 shows examples of these phasors; by simply changing the phase offset of the applied Bloch-Siegert pulse, distinct phase maps with varying amounts of phase winding are created. Figure 2B shows the phasor pattern when transmitting from eight out of the nine encoding coils, with all pulses applied in phase. Figure 2C shows the change in phasor pattern when those same eight coils are selected for transmit, but the pulse applied to the middle row of coils is $\pi$ out of phase^32^. These unique phasors assign signature phase evolutions to each voxel in the image, similar to phase encode or readout gradients in conventional MRI. For clarity, in this paper we will refer to “encoding” as a single k-space point and “projection” as a set of encoding points which is equivalent to a k-space trajectory.

### Nonlinear encoding scheme design

Parallel imaging using nonlinear encoding fields introduces many degrees of freedom in encoding scheme design; encoding patterns may vary with time, they exhibit higher orders of spatial variations compared to linear gradients and are often only limited by the hardware available for encoding. With our low-field open MR system, encoding patterns are dependent upon the number of coils selected for encoding, and the phase of each of those coils, as described in the previous section. This then produces thousands of possible encoding schemes, but to improve encoding efficiency we need a scheme that provides the most spatial information with minimal repeated information.

Traditional encoding analysis tools look at the relationship between the data and k-space^33^. However, this formulation does not hold for nonlinear encodings because k-space varies spatially in the image domain and local k-space analyses are needed. There are methods to quantify the coverage of k-space with nonlinear gradients in parallel imaging^34^ and characterize their performance^35^, but these consider entire encoding trajectories and cannot be easily translated for building optimized projection-based trajectories. Designing encoding schemes with nonlinear RF gradient fields requires new, robust optimization techniques that will analytically evaluate the uniqueness and amount of spatial information provided by an encoding pattern.

**Figure 3:**
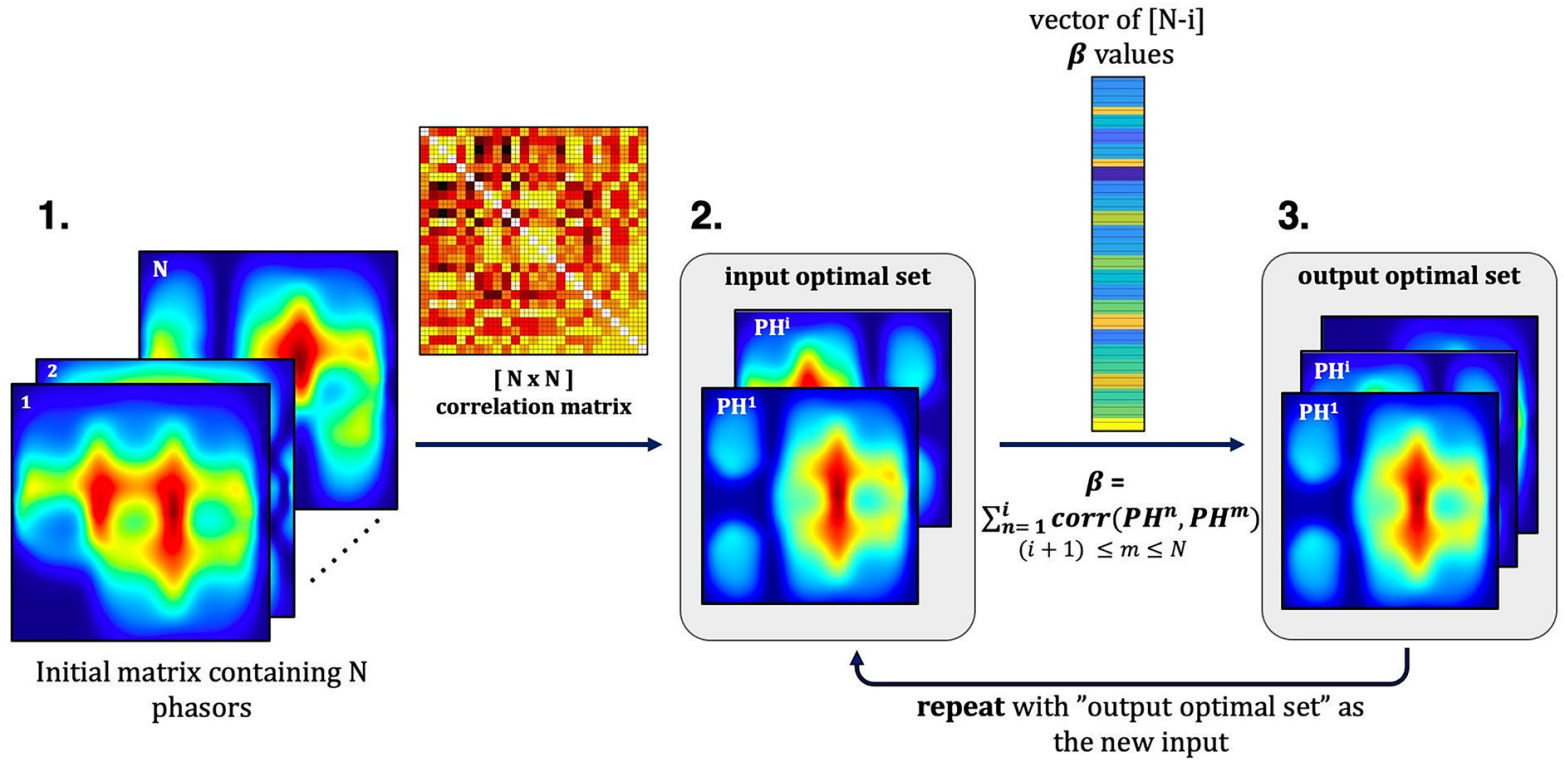
Encoding scheme design: The correlation between pairs of phasors was calculated for the initial set of N phasors (1). The pair of phasors with the smallest correlation was added to the optimal set (2). The sum of correlations squared was calculated between the remaining (N − i) phasors and the phasors in the optimal set, for a total of (N − i) $\beta$ values. The phasor with the smallest $\beta$ value was then added to the optimal set (3). The “output optimal set” became the new input, and step 3 was repeated until the final optimal set contained the desired number of phasors.

Figure 3 describes the method used in this study to design optimized encoding schemes. We separated sets based on the number of coils used to transmit the Bloch-Siegert pulse and limited the phase variations ($$\varphi$$ in Eq. 6) to 0 and π. Phase offsets of 0 or π were selected because they resulted in encoding patterns that were most dissimilar from one another, as seen in the example presented in Fig. 2. To evaluate these phasors, we first unwrapped the phase of each voxel over time which reveals the frequency evolution at each point. This is equivalent to looking at the extent of k-space coverage by each encoding pattern. From these unwrapped phasors, we calculated the L2 norm in a 20 cm × 20 cm region in the center of the FOV (the target imaging area). A large L2 norm in the center of the FOV correlates with a strong B_1_ magnitude field and high encoding efficiency. We then selected a subset of *N* phasors (top 30%) with the highest L2 norm and calculated the correlation between pairs of phasors ($$PH)$$ in this subset. The pair with the smallest correlation was added to a new empty set that will be referred to as the “optimized set”. Next, the sum of correlations squared was calculated between the remaining *N − i* phasors and the phasors in the optimized set to generate a total of (*N − i*) *β-*values where *β* is defined as:

7 $${\beta }_{m}=\sum_{n=1}^{i}{corr\left({PH}^{n},{PH}^{m}\right)}^{2}, \left({\text{i}}+1\right)\le m\le N$$

In Eq. (7), *i* is the total number of phasors in the chosen set, and *m* is the index of the phasor in the remaining set (*N − i*). For the first iteration *i* = 2. The phasor with the smallest *β-*values was then added to the optimized set. This process was repeated recursively until the final chosen set contained the desired number of phasors. This pattern selection technique ensures that the selected phasors are maximally different from each other while providing spatial encoding over the desired FOV.

### Signal simulation and reconstruction

The NMR signal acquired by each receiver channel for the *i*-th encoding field was modeled with the following equation:

8 $${s}_{r}\left(x,y,\tau \right)= \int \rho \left(x,y\right)*{C}_{r}\left(x,y\right)*{e}^{{i*\phi }_{BS}\left(x,y,\tau \right)}dxdy$$

where $\rho \left(x,y\right)$ is the object being imaged (phantom), ${C}_{r}\left(x,y\right)$ is the coil sensitivity profile in complex form for the rth receive channel, and ${\phi }_{BS}\left(x,y,\tau \right)$ is the phase evolution in radians over the duration of the applied Bloch-Siegert pulse $(\tau )$ for a single encoding pattern (Eq. (4) with B1 = B1_E_). A spin-echo sequence was simulated with a TE = 16 ms, T1 = 3000 ms, and T2 = 250 ms. Instead of conventional readout gradient, the off-resonance Bloch-Siegert pulse was applied for 6 ms with a sampling rate of 0.023 ms, for a total of 256 samples.

Since the encoding fields produced by the RF array coils are nonlinear, Fourier transform-based image reconstruction techniques are not suitable. This is because the nonlinear fields create readout signals that contain both k_x_ and k_y_ information, which causes non-equidistant k-space filling, as well as spatially dependent local k-space trajectories^36^. Therefore, we used the conjugate gradient descent method for algebraic reconstruction^24,26,34,37^ to solve the matrix inverse problem. Equation (8) can be rewritten as:

9 $$\overset{\lower0.5em\hbox{$\smash{\scriptscriptstyle\rightharpoonup}$}}{{\text{s}}} _{{r,p,t}} = E_{{r,p,t}} \overset{\lower0.5em\hbox{$\smash{\scriptscriptstyle\rightharpoonup}$}}{\rho } = C_{r} B_{{p,t}} \overset{\lower0.5em\hbox{$\smash{\scriptscriptstyle\rightharpoonup}$}}{\rho }$$

where $r,p$ and $t$ are index numbers indicating the selected receiver coil, projection, and readout points, respectively. ${{\text{E}}}_{r,p,t}$ is the known encoding matrix that contains both the coil sensitivity profiles ($C$) as well as the encoding field pattern ($B$), and $\mathop{\rho }\limits^{\rightharpoonup}$ is the true image that is being solved for with iterative conjugate gradient minimization. Images of a grid phantom and a high resolution 3 T liver image were reconstructed in simulations using 32 or 128 projections (analogous to k-space trajectories) with 256 readout points per echo, for a matrix size of 256 × 256 over a 40 cm × 40 cm FOV.

### Experimental study

**Figure 4:**
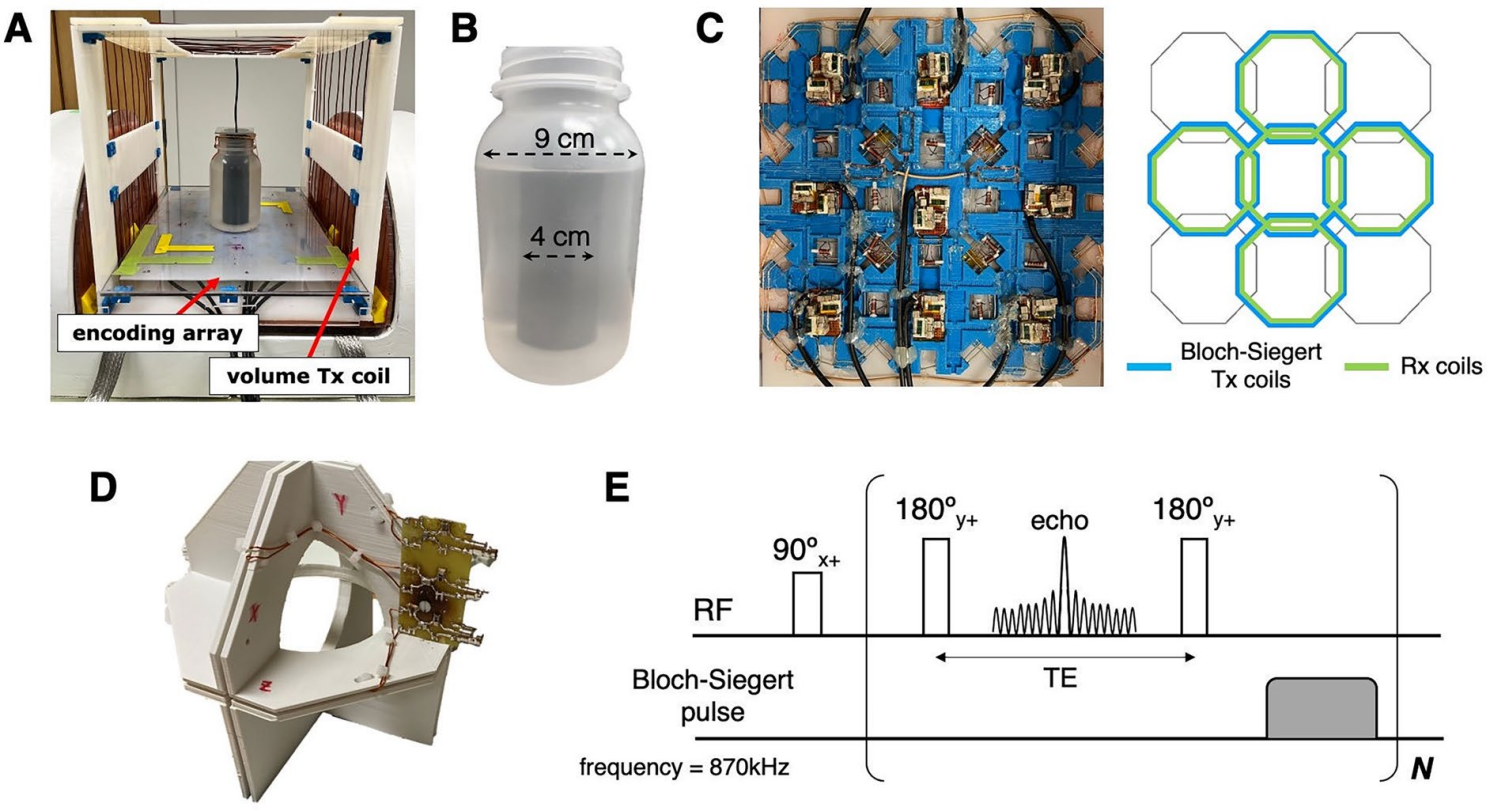
Experimental setup. (**A**) Experiments were conducted using a large Tx-only volume coil to transmit the excitation and refocusing pulses. The RF encoding array was positioned at the base of the magnet, and the phantom was placed on top of the array in the center of the FOV. (**B**) The large water-filled bottle phantom with a diameter of 10 cm that was imaged. A 4 cm solid plastic rod was placed in the center of the water bottle to create an imaging feature. (**C**) Photograph of the array coils used for spatial encoding. Each element in the array is switched tuned to an off-resonance frequency (870 kHz) to transmit the high-power Bloch-Siegert pulse, and the resonance frequency (1 MHz) to receive the NMR signal. For initial experiments five channels, indicated in blue and green, were used to create the encoding patterns and receive the resulting signal. (**D**) Picture of the 3-axis probe that was set adjacent to the magnet to detect the EMI signal. (**E**) The multi-echo CPMG pulse sequence used in experiments. The sequence shown leads to positive k-trajectory traversal, with the Bloch-Siegert pulse applied to even echoes and odd echoes are collected. For negative k-trajectory traversal, the Bloch-Siegert pulse was applied to odd echoes and even echoes were collected.

Imaging experiments were conducted using the NuBo magnet^38^, a low-field, single-sided rampable electromagnet system to validate the performance of the encoding scheme optimization technique. The experimental setup consisted of a large Tx-only volume coil set inside the shielded magnet to transmit the 90 and 180 pulses, an RF encoding array positioned at the base of the magnet, and a water-filled bottle phantom with a diameter of 9 cm placed in the center of the FOV (Fig. 4A). The phantom contained a 4 cm solid plastic rod located in the center of the bottle (Fig. 4B). The RF encoding array is shown in Fig. 4C. Due to constraints in available hardware, only the five channels indicated in blue were used to transmit the Bloch-Siegert pulse and for parallel receive (green). We will refer to the encoding coils used in experiments as the “5-channel array”.

Each element in the 5-channel array is switched tuned to both the off-resonance Bloch-Siegert frequency (870 kHz), and the on-resonance receive frequency (1 MHz). The coils are designed such that when a high-power RF pulse is applied, the coil matching network is tuned to 870 kHz; in all other instances the coil tuning remains at 1 MHz. Encoding patterns were generated by selecting combinations of 1, 2, 3 or 4 coils to transmit the Bloch-Siegert pulse, where the pulse to each coil was applied with a phase of 0 or $\pi$. The pulse was applied such that the magnitude of the resulting B_1_-field was at least one order of magnitude lower than the off-resonance, thereby meeting the condition stipulated in Eq. (1). Two different encoding schemes were tested: (1) an optimized set, and (2) a low L2 norm set that would be excluded from the first module of our optimization scheme. With 5 transmit channels the total number of unique encoding patterns that can be generated is limited. To ensure no overlap between the optimal and small L2 norm sets, each contained a maximum of 32 and 24 projections, respectively.

Electromagnetic interference (EMI) was mitigated in the acquired signal using a modified version of the EDITER technique^39^. The 3-axis probe shown in Fig. 4D was placed adjacent to the magnet and was used to detect EMI signal. Similar to EDITER, we assumed a linear convolution model between the EMI observed by the primary imaging coils and the external detectors. However, unlike EDITER the impulse response function coefficients were determined using calibration data acquired from a pre-scan conducted with the imaging pulse sequence (Fig. 4E), but with no phantom.

Figure 4E shows a diagram of the multi-echo encoding CPMG pulse sequence used in experiments. In this sequence the Bloch-Siegert RF pulse acts much like a blipped phase encode gradient in conventional turbo spin echo imaging. With this pulse sequence, the positive k-space trajectory is acquired within one TR. TR is rate limited by the polarization period where B0 is at its maximum; this was set to 6 s. To obtain the negative k-space trajectory, the Bloch-Siegert pulse was applied on odd echoes and even echoes were acquired. 400 were collected over the entire k-trajectory with an echo spacing of 2 ms, and a total of 160 averages. Each echo peak in the echo train is analogous to a single k-space readout point. The Bloch-Siegert pulse blips were applied with a constant frequency offset, and duration of 350 us. Spatial encoding B_1_-field maps for image reconstruction were estimated using Eq. (6). Images were reconstructed using these B_1_-field maps, over a 20 cm × 20 cm FOV with a matrix size of 64 × 64.

## Results

**Figure 5:**
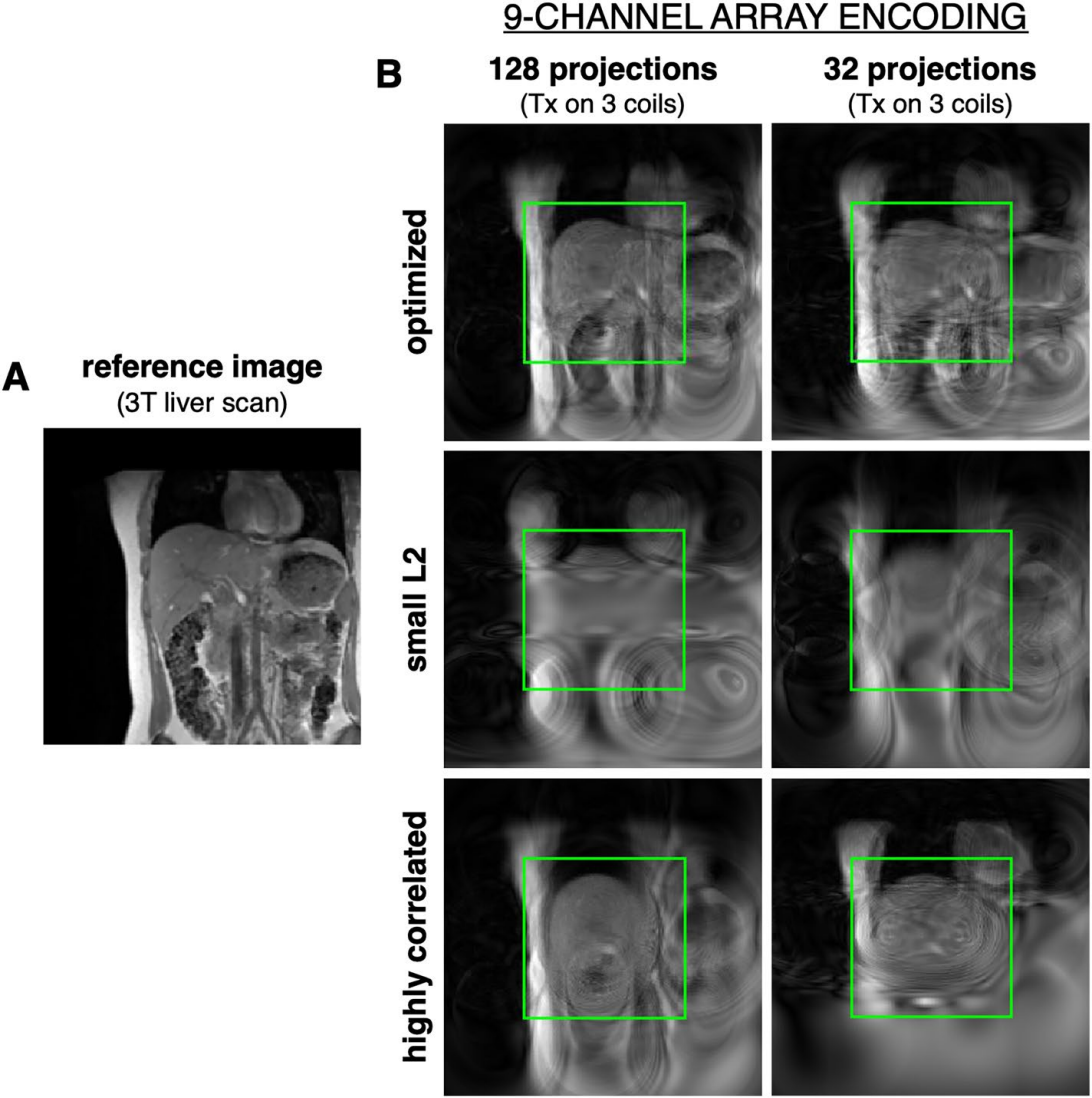
Optimization performance. (**A**) Reference liver image used in simulations. (**B**) Liver images reconstructed using an optimal (top row), small L2 norm (middle row) and highly correlated (bottom row) set of 128 and 32 projections. All encoding patterns were generated from transmitting the Bloch-Siegert pulse on 3 coils on the 9-channel RF array. The green box indicates a 20 cm × 20 cm FOV.

The performance of the optimization algorithm was assessed through simulations of encoding patterns created from the 9-channel array, and acquisition using the reference image shown in Fig. 5A. This liver image is of a nonplanar slice extracted from a high-resolution volume 3 T liver scan. Figure 5B shows the image quality obtained by an optimal encoding scheme (top row) of 128 and 32 projections from transmitting the Bloch-Siegert pulse on different combinations of 3 coils—denoted as “Tx on 3 coils” in Fig. 5. Note that the image quality is best within the center 20 cm × 20 cm FOV, which is the region targeted by our optimization strategy. Since the primary features of our optimization method consists of choosing phasors with a high L2 norm in the center FOV and minimal correlations, we also tested encoding schemes that violate these conditions. Figure 5B middle row shows the reconstructions from projections that had the lowest L2 norm in the center of the FOV. As expected, these images have poor resolution and appear very blurred. The bottom row of Fig. 5B shows the reconstruction results from sets of 128 and 32 projections that meet the L2 norm criteria but are highly correlated. Particularly for a set of 32 projections, it can be seen that encoding with this set is less efficient and gives stronger undersampling artifacts. When choosing 128 projections, small correlation among encoding patterns becomes inevitable even without optimization. However, this encoding scheme still does not perform as efficiently as the optimized set.

**Figure 6:**
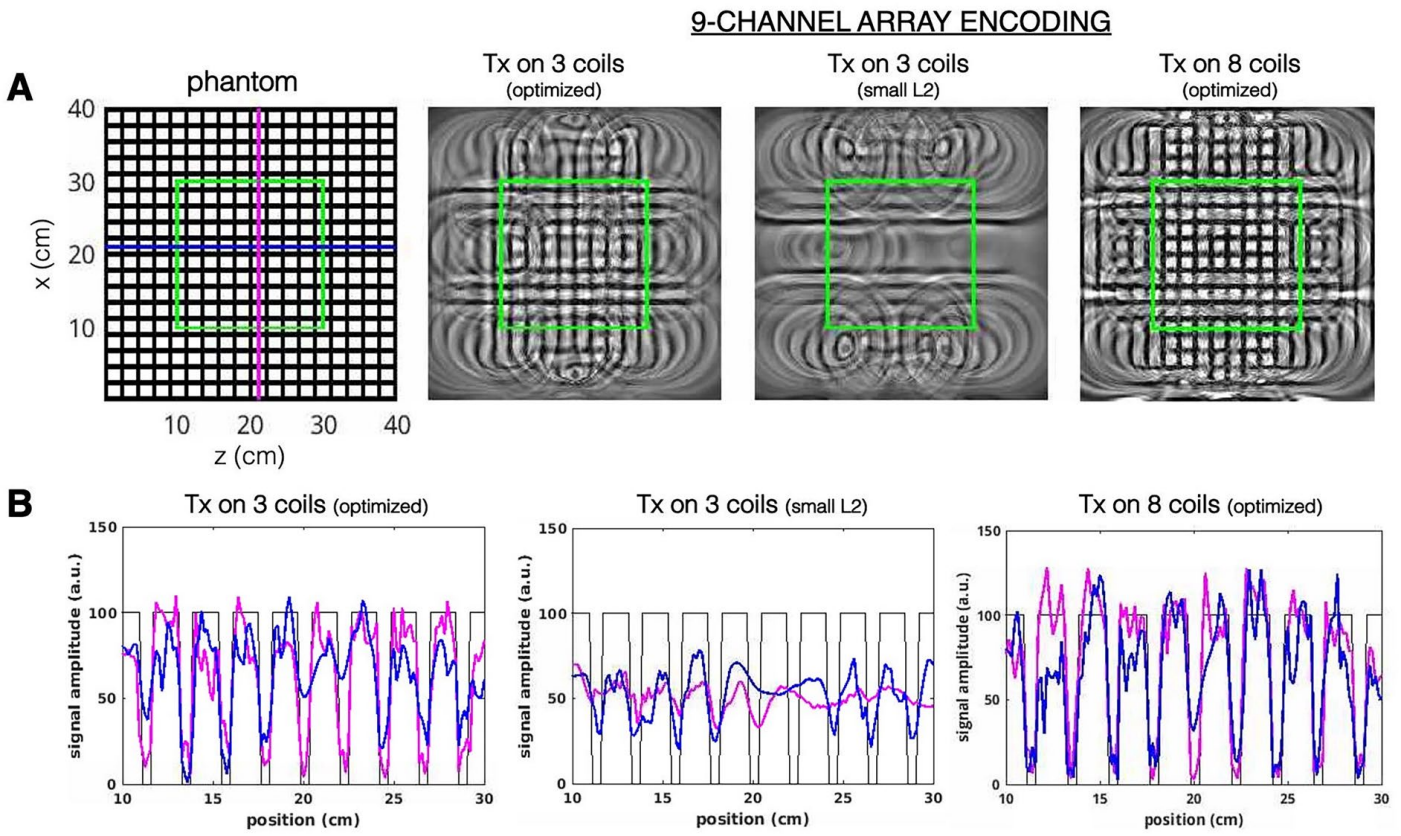
Grid phantom reconstruction. (**A**) Reconstruction results of a grid phantom image encoded using an optimal set of phasors created from transmitting the Bloch-Siegert pulse on 3 coils, a small L2 norm set from Tx on 3 coils and an optimal set from Tx on 8 coils (left to right). Each encoding scheme contained a total of 128 projections. The green box indicates a 20 cm × 20 cm region of interest. (**B**) Plots showing the signal profile within the center 20 × 20 cm FOV across a horizontal (blue) and vertical (pink) line for each encoding scheme. The black line is the ideal signal profile of the phantom image.

To better visualize the spatial resolution limitations of the optimization technique, and its tolerance to spatial distortions, simulations were conducted on grid phantom images. Image reconstruction was performed with 128 projections over a 40 cm × 40 cm FOV with a matrix size of 256 × 256. Figure 6A shows the reconstruction result from an optimized encoding scheme from transmitting the Bloch-Siegert pulse on various combinations of 3 coils. Visual inspection shows that the image quality is best within the center 20 cm × 20 cm FOV, with a structural similarity index measure (SSIM) = 0.32. Line plots of the signal profile in the center 20 cm × 20 cm FOV along the horizontal (blue) and vertical (pink) lines are shown in Fig. 6B. The blue and pink line profiles in the optimized encoding schemes in Fig. 6B more closely match the ideal signal profile shown in black. In contrast, reconstructions using a low L2 norm set, which would be excluded from our optimization show low resolution. While encoding schemes generated from transmitting on 3 coils better correspond to what is experimentally achievable with our current 5-channel array, the 9-channel RF array imparts more flexibility in designing encoding patterns. Figure 6A also shows the reconstruction from an optimized set of encodings with transmit on 8 channels (SSIM = 0.5). Qualitative assessment of the center 20 cm green box shows good fidelity for the highly challenging features of this phantom and the corresponding line plots illustrate the improvements in resolution in both the x and z-directions.

**Figure 7:**
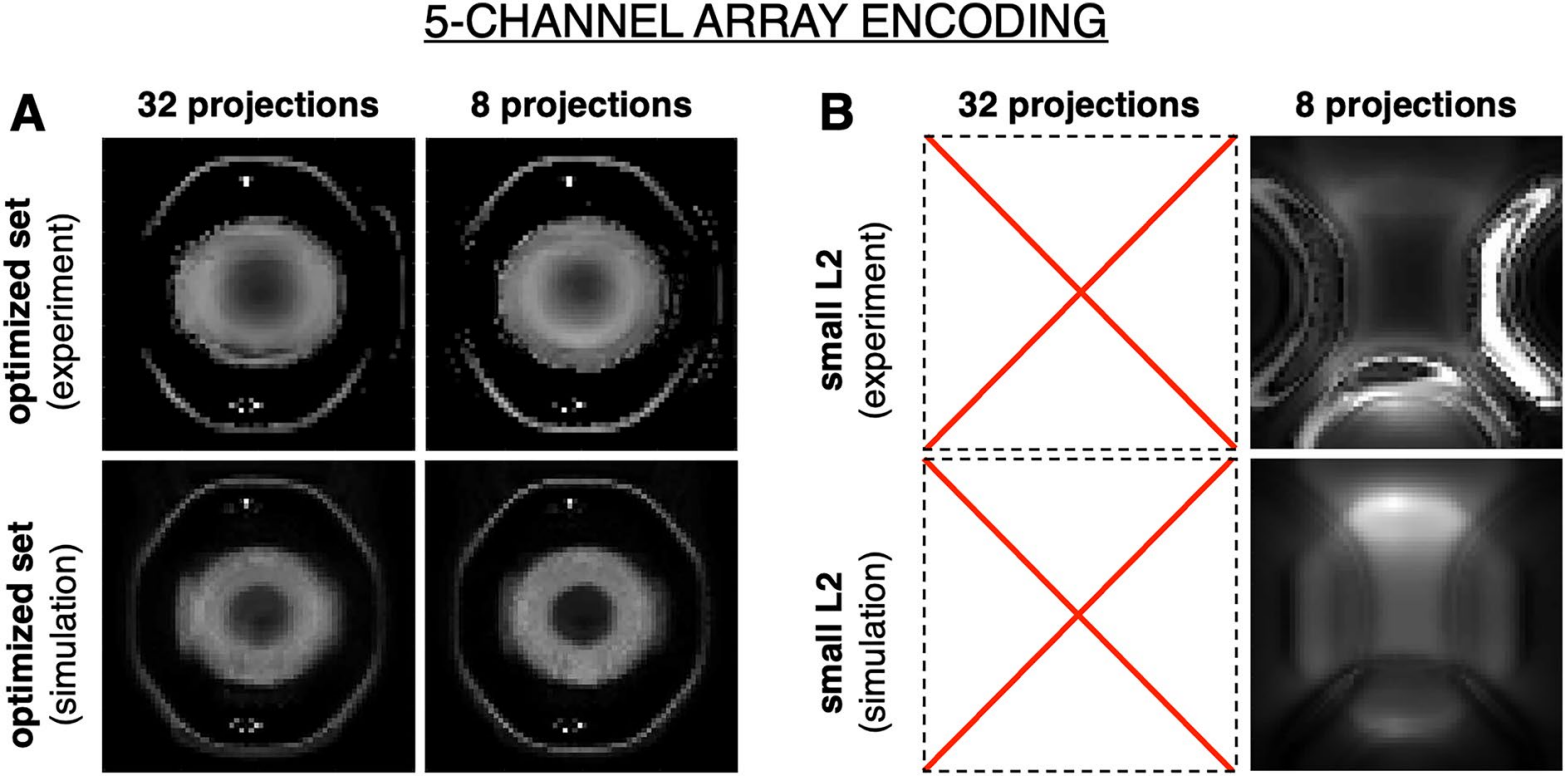
Experimental results. Experimental and simulation results from imaging a 9 cm diameter phantom with a 4 cm solid rod placed in the center. Results are shown for (**A**) optimized sets of 32 and 8 projections, and (**B**) a small L2 norm set of 8 projections. Image reconstruction was performed over a 20 cm × 20 cm FOV, with a 64 × 64 matrix.

Figure 7A shows the experimental results from imaging a hollow water bottle phantom using the 5-channel array with a set of phasors chosen by our optimization algorithm. For these experiments, images were reconstructed with 32 and 8 projections. As can be seen, the set optimized for encoding efficiency provides similar image quality for the larger and smaller number of projections, though some features are better delineated with additional projections. In addition, experimental results show good agreement with those predicted by simulation.

Similar to the results presented in Figs. 5 and 6. Imaging with the same number of projections but using patterns with low L2 results in very low resolution reconstructions (Fig. 7B). Note that, given the limited number of encoding patterns available with the 5-channel array, it was not possible to identify 32 phasors with low L2 norm.

**Figure 8:**
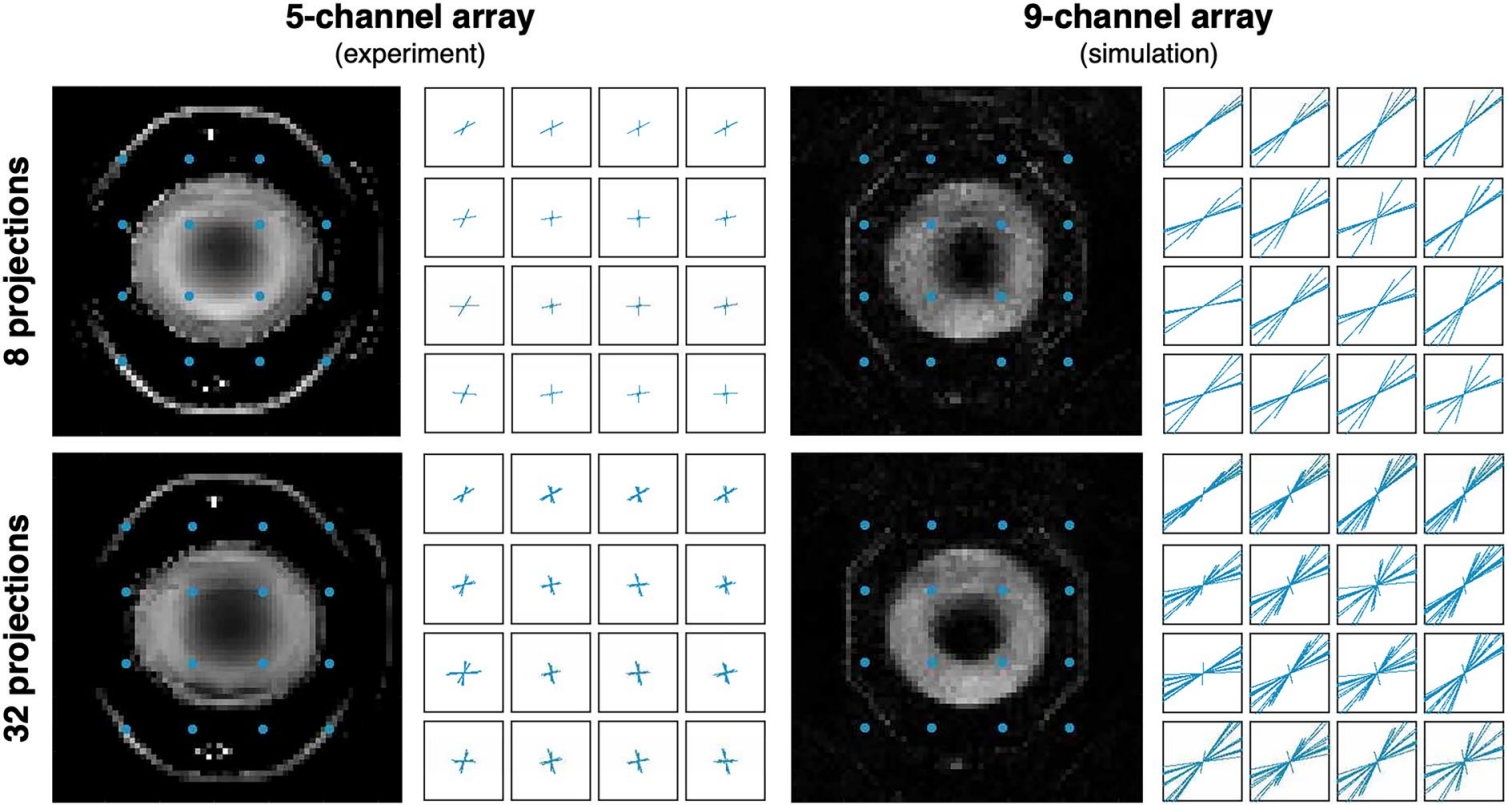
Local k-space analysis. Left column: Images reconstructed from experimental data collected on the 5-channel array, with optimized sets of 8 and 32 projections. The local k-space is shown for the points indicated in blue. Right column: Simulated image reconstructions and corresponding local k-space for optimized set of 8 and 32 projections on the 9-channel array*.* Encoding with the 9-channel array and using a large number of Bloch-Siegert transmit coils increases k-space coverage and image resolution.

The marginal improvement in reconstruction quality between the optimized set of 8 and 32 projections from the 5-channel array can be explained by examining the local k-space. Whereas with conventional linear gradients the k-space can be visualized as a single 2D graph, with nonlinear encodings the k-space coverage is variable for every pixel in the image. Figure 8 shows the local k-space for 16 locations in the FOV. While increasing the number of projections begins to fill in some of the gaps in k-space, encoding patterns generated from the 5-channel array are unable to cover the full extent of k-space (Fig. 8, top row). However, encoding with the 9-channel array using projections created from transmitting the Bloch-Siegert pulse on 8 coils increases k-space coverage, as is reflected in the improved image quality of the simulated image (Fig. 8, bottom right).

## Discussion

In this study, we introduce a novel optimization method for nonlinear encoding schemes that uses RF spatial encoding exploiting the Bloch-Siegert shift. Through simulation studies and preliminary experiments, we were able to robustly design and assess different nonlinear encoding schemes and evaluate their performance through image reconstruction. Spatial encoding using the Bloch-Siegert shift is generally inefficient because it is applied off-resonance and as such the specific absorption rate (SAR) limits its application at high field. Since SAR scales with B_0_^2^^40^, this technique is well suited for spatial encoding at lower field strengths. Previous work on RF encoding systems rely on the conventional linear B_0_-gradients for at least one of the encoding directions for imaging^13,17,18^; even studies involving the Bloch-Siegert shift for spatial encoding utilized it in combination with conventional linear gradients. These studies also kept to linear B_1_ encoding fields and imaged over a small FOV^18,19^. In contrast, we present a completely novel approach to 2D spatial encoding with the Bloch Siegert shift by implementing a strategy that embraces the inherent nonlinearity of the B1-fields generated by planar RF array coils. Moreover, since the only priors required for image reconstruction are the field maps of the encoding fields, this technique can be translated to RF array coils of any shape, size, or geometry, and can be used to image over any FOV.

Imaging with nonlinear RF encoding fields introduces many degrees of freedom; including which RF coils are used for transmit and their relative phases and amplitudes. While this flexibility may have the potential for highly efficient encoding, there is no clear framework for designing an efficient and complete set of encoding trajectories for image reconstruction. In general, appropriate imaging schemes using nonlinear encodings are challenging, particularly in 2D, because the encodings can only be described using local k-space. For all nonlinear encodings, image resolution and k-space coverage are variable over the FOV. Prior work studying nonlinear encoding with gradients have mostly focused on understanding the relationship between nonlinear SEMs, k-space coverage and image reconstruction performance^34–36^, and less on encoding scheme design. Tam et al. has presented a strategy to design gradient encodings for accelerated imaging based on the null space of receiver coils^41^, and Layton et al. has proposed a method to optimize encoding trajectories based on the covariance of reconstructed pixels which is inversely proportional to image SNR^42^. However, these methods posit a small number of available encoding shapes that can be linearly combined or temporally varied. In contrast, for RF encoding, the fields do not add linearly, and the number of potential shapes is orders of magnitude greater, making such a recursive optimization approach computationally expensive.

Our optimization technique has some similarities to this previously proposed approach, but instead of estimating the magnetization image from each projection we directly analyze the relationship between the encoding field and image resolution. Image resolution with Bloch-Siegert encoding is related to the magnitude of the ${B}_{1}$-field and the extent of spatial variations in the encoding patterns: resolution improves when the number of pixels that map to a particular encoding-field is minimized. The optimization algorithm developed in this study accounts for the former (B_1_-field magnitude) by selecting phasors with the highest L2 norm in the center of the FOV, and accounts for the latter (field variations) by selecting phasors for the final encoding scheme that are minimally correlated with each other.

The simulation results in Fig. 5 show that encoding schemes designed using our optimization technique produces better liver image reconstructions compared to “anti-optimized” encoding schemes for sets with 32 and 128 projections. Simulations using the same 128 projections with Tx on 3 coils but applied on a grid phantom also showed similar results. This was verified experimentally with a hollow water bottle phantom. Images encoded with an optimized set of projections outperformed that of a small L2 norm set (Fig. 7). Furthermore, it is notable that the image quality observed with 32 projections was similar to that seen with 8 projections, which suggests good efficiency of the optimized set. It should be noted that the proposed approach is heuristic and does not guarantee optimality, though results indicate that the suggested criteria do improve image quality. Previous studies have considered more generalized metrics of encoding performance, such as those based on frame theory^35^ but these do not easily lend themselves towards identifying an optimal encoding, particularly over a high dimensionality search space. We have also studied data compression of the encoding matrix, but such methods revealed rapidly decaying singular values, which suggests further encoding improvements are needed^43^.

The experimental reconstructions performed in this study were done using simulated encoding field maps. While the coil geometry and imaging slice location are known based on prior measurements, they are still subject to human errors. Future work will focus on using experimentally measured B_1_-field maps in image reconstructions to avoid any uncertainties in slice locations and field patterns. This will further help improve our image quality.

Reconstruction results shown in Fig. 8 also highlight the importance of RF geometry in achievable image quality. With the 9-channel array, significant image quality improvements could be seen with additional projections, especially when those projections are optimized for maximal efficiency. In contrast, with the 5-channel array, image quality improvements between 8 and 32 projections were considerably more subtle, both in experiment and simulation. This is because the span of possible projections is much more limited when transmit is restricted to fewer channels, so 8 projections are nearly sufficient to capture the variability that can be achieved with this hardware, especially over the small field of view used in experimental studies.

The importance of coil geometry is also evidenced by our local k-space analysis (Fig. 8). The 5-channel array has large gaps in local k-space and minimal coverage of the edges of k-space which results in the ring artifact seen in the images. This artifact is a result of the encoding fields in those regions having frequency isocontours similar to the ones in the phantom area. These artifacts are significantly reduced when encoding with the 9-channel array (Fig. 8). The 9-channel array is able to cover a larger extent of k-space and produce encoding fields with unique frequencies at various spatial locations which results in improved image quality with minimal artifacts. Simulations suggest that the current geometry of the 9-channel array coil can achieve resolution ~ 1 mm over the central FOV, albeit with residual artifacts due to the uneven sampling of angular k-space. It is important to note that this work studies only optimization of projection-based encoding strategies. Other groups have shown progress incorporating phase encoding or arbitrary readout trajectories into nonlinear encoding scheme^36,44,45^. But the narrow nature of local k-space generated from projections with the current hardware system indicates that a trajectory designed to reach the gaps in a given local k-space would necessarily be somewhat inefficient, and achieving this for all voxels would introduce further encoding inefficiencies. Therefore, this further generalization of the encoding trajectory was not pursued in this work. Still, early studies on other strategies, such as spatial shifting of the RF array between echoes, suggest that improved sampling of local k-space is possible^46^. It is an open question whether alternative RF geometries could further improve resolution and artifacts.

The experiments in this work were conducted using a multi-echo pulse sequence where the Bloch-Siegert RF pulse acted like a blipped phase encode gradient, thereby encoding one point in k-space with each echo; this renders scan time on the order of hours. While this method is good for demonstrating proof-of-concept, for practical applications simultaneous transmit of a high-power Bloch-Siegert off-resonance pulse and receive on-resonance is necessary. The realization of both frequency and phase encoding with the Bloch-Siegert shift requires further developments in coil tuning/matching networks, T/R switch design and signal filtering^47^. Accounting for averages and repeated polarization, this would result in an acquisition times of 5–6 min.

Finally, we note that these proof-of-concept studies made use of a birdcage coil for excitation and refocusing, which would not be suitable for open body imaging. With appropriate B1 shimming, it should be possible to achieve sufficiently uniform excitation and refocusing across the slice using the planar array. Alternatively, one could envision a split bird-cage that fits around the torso, which many patients may find more tolerable than existing whole-body geometries.

## Conclusion

In this study we have designed and analyzed novel nonlinear RF encoding schemes for 2D gradient-free imaging at low-fields. We propose a technique to design 2D encoding trajectories and we validated its performance through simulation and experimental studies. The results showed that the optimal encoding schemes produce images with increased resolution and reduced artifacts compared to non-optimized encoding schemes. Overall, the work presents a method to design efficient nonlinear encoding schemes and demonstrates the feasibility of using these schemes to perform nonlinear RF spatial encoding and 2D imaging with the Bloch-Siegert shift which could help make MRI lower cost, silent, and highly accessible.

## Data Availability

The datasets generated and analyzed during this study are available from the corresponding author on reasonable request.
